# Loading of Single Atoms of Iron, Cobalt, or Nickel to Enhance the Electrocatalytic Hydrogen Evolution Reaction of Two-Dimensional Titanium Carbide

**DOI:** 10.3390/ijms25074034

**Published:** 2024-04-04

**Authors:** Kaijin Wang, Jing Yu, Qi Liu, Jingyuan Liu, Rongrong Chen, Jiahui Zhu

**Affiliations:** Key Laboratory of Superlight Materials and Surface Technology, Ministry of Education, College of Materials Science and Chemical Engineering, Harbin Engineering University, Harbin 150001, China; 1224656345@hrbeu.edu.cn (K.W.); qiliu@hrbeu.edu.cn (Q.L.); liujingyuan1004@hrbeu.edu.cn (J.L.); chenrongrong@hrbeu.edu.cn (R.C.); jiahuizhu@hrbeu.edu.cn (J.Z.)

**Keywords:** titanium carbide, transition metals (Fe, Co, or Ni), single-atom catalysts, molten salt method, hydrogen evolution reaction

## Abstract

The rational design of advanced electrocatalysts at the molecular or atomic level is important for improving the performance of hydrogen evolution reactions (HERs) and replacing precious metal catalysts. In this study, we describe the fabrication of electrocatalysts based on Fe, Co, or Ni single atoms supported on titanium carbide (TiC) using the molten salt method, i.e., TiC-Fe_SA_, TiC-Co_SA_, or TiC-Ni_SA_, to enhance HER performance. The introduction of uniformly distributed transition-metal single atoms successfully reduces the overpotential of HERs. Overpotentials of TiC-Fe_SA_ at 10 mA cm^−2^ are 123.4 mV with 61.1 mV dec^−1^ Tafel slope under acidic conditions and 184.2 mV with 85.1 mV dec^−1^ Tafel slope under alkaline conditions, which are superior to TiC-Ni_SA_ and TiC-Co_SA_. TiC samples loaded with transition-metal single atoms exhibit high catalytic activity and long stability under acidic and basic conditions. Density functional theory calculations indicate that the introduction of transition-metal single atoms effectively reduces the HER barrier of TiC-based electrocatalysts.

## 1. Introduction

Due to their high energy density, fossil fuels such as coal, oil, and natural gas have been exploited as major energy sources; however, the negative impacts of fossil fuel combustion on water acidification, air pollution, and the greenhouse effect cannot be ignored [1,2]. Consequently, there is growing interest in finding more efficient, clean, and sustainable alternative energy sources. Contextually, hydrogen energy, the “ultimate energy source”, stands out owing to its high energy density and environment friendliness [3]. Hydrogen can be prepared via diverse methods such as steam methane reforming, coal gasification, and water electrolysis [4,5]. In contrast, the reaction of methane or coal at high temperatures leads to CO_2_ byproducts and concomitant environmental pollution [5,6]. Water splitting, as a reverse reaction of hydrogen combustion, is considered one of the most promising hydrogen preparation methods concerning its economic and environmental advantages [7,8,9,10]. To improve the efficiency of the hydrogen evolution reaction (HER) and reduce the power consumption, using active electrocatalysts to reduce HER overpotential is crucial [11]. Precious metal-based materials have excellent HER performance. The modification with transition-metal-based materials could help to lower the use of precious metals by keeping the high HER activity [12,13]. Even so, the high price and scarcity greatly limit the widespread application of precious metals in electrocatalyst HERs [14]. Finding a more economical, efficient, and stable HER electrocatalyst is essential. Transition metals and their compounds show great potential in water splitting [15]. In particular, transition metals such as Fe, Co, and Ni have the potential to be used as HER electrocatalysts due to their large reserves, low cost, and optimized electronic structure.

With the increasing research on multiphase catalysts, the construction and research of nano and sub-nano active sites at the molecular level have become increasingly important. Single-atom catalysts (SACs) have become promising candidates in the field of catalysis due to their unique properties [16]. Unlike metal nanoparticles, SACs are atomically dispersed and coordinate unsaturated metal active sites, with a theoretical atom utilization efficiency of 100% [17]. In addition, SACs have a uniform distribution of active sites, greatly increasing the number of highly active sites [18]. In addition, dispersed SACs can lead to localized electronic structure adjustments, exhibiting higher activity, stability, and selectivity, thus effectively increasing the catalytic activity [19]. Compared with traditional loaded catalysts, SACs require lower loadings and exhibit higher energy conversion rates, thus effectively reducing costs and improving economic efficiency. However, the high surface free energy of SACs renders them prone to migrate and aggregate, eventually forming nanoparticles [20]. Therefore, the selection of suitable substrates to achieve a more uniform dispersion of single atoms is important for efficient catalytic performance.

Transition-metal-based materials, including transition metal carbides, nitrides, sulfides, and phosphides, are gaining interest in areas such as catalysis and energy storage [21,22,23,24]. Among them, transition-metal-carbides have been widely studied in the electrocatalytic splitting of water due to their noble-metal-like electronic properties, excellent physical properties, good electrical conductivity, and stability [25,26]. Particularly, titanium carbide (TiC), exhibiting a typical face-centered cubic crystal structure [27], offers the advantages of high hardness, high melting point, corrosion resistance, and good electrical and thermal conductivity [28]. Among the available methods for TiC synthesis, direct carbonization of solid-phase Ti powder is the most straightforward method [29]. However, it suffers from high reaction temperature, long reaction times, and inhomogeneous particle size of the resulting products. Meanwhile, the carbothermal reduction in solid-phase TiO_2_ requires high-temperature conditions and results in more oxygen-containing impurities in the product [27]. Moreover, TiC can be prepared via chemical vapor deposition by thermally decomposing carbon-containing gases such as CH_4_ and C_2_H_6_ followed by the reaction with TiO_2_ [30]. Unfortunately, this method consumes a large amount of fossil fuels, and the produced TiC is easily contaminated with impurities generated by gas pyrolysis, resulting in reduced catalytic activity [31,32]. The preparation of lamellar TiC via sublimation and volatilization of A-layer in M_n+1_AX_n_ phases (MAX, where M stands for transition metal, A for III A or IV A element, generally Al, X for C or N [33,34]) at high temperature is still relatively rare. Sublimation at high temperatures removes the A-layer, resulting in TiC nanosheets with a lamellar structure. Chemical loading with heteroatoms has been widely investigated to optimize HER catalytic performance by adjusting the electronic properties and elemental composition [35]. Particularly, loading transition-metal single atoms into TiC defects is a very effective strategy to achieve high catalytic activity.

In this study, single-atom-loaded TiC electrocatalysts were synthesized via high-temperature calcination of the MAX phase of Ti_2_ZnC using NaCl-KCl mixed molten salts followed by vapor deposition. The results show that the introduction of transition-metal atoms (Fe, Co, or Ni) considerably increases the electron transfer rate of TiC and improves its electrocatalytic performance. Overpotentials of Fe-, Co-, or Ni-loaded TiC at 10 mA cm^−2^ current density under acidic and alkaline conditions are improved compared with pristine TiC. In addition, TiC-Fe_SA_ shows the best electrocatalytic performance under acidic and alkaline conditions with the highest mass activity and turnover frequency (TOF). Density functional theory (DFT) calculations show that the introduction of Fe, Co, or Ni into TiC favors the adsorption of water and hydrogen, suggesting that the transition-metal loading, particularly with Fe, enhances HER performance.

## 2. Results and Discussion

### 2.1. Structural Characterization

**Figure 1:**
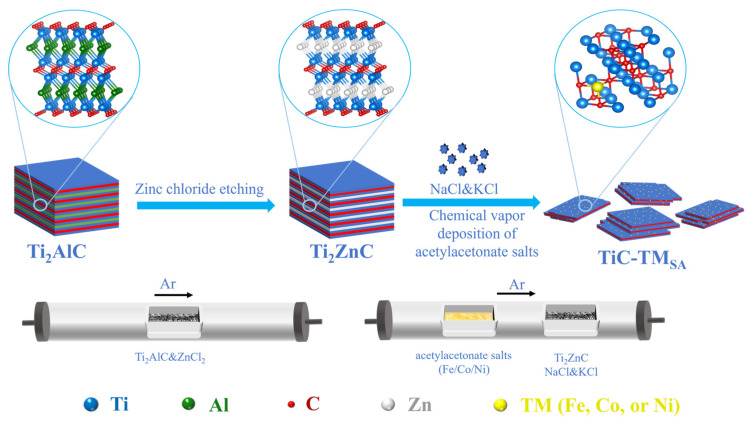
Schematic of preparation of TiC loaded with Fe, Co, or Ni single atoms.

Homogeneously dispersed TiC-based electrocatalysts loaded with transition-metal single atoms were prepared via high-temperature calcination of the Zn-MAX phase using the molten salt method under the Ar atmosphere (Figure 1). The Zn layer was removed from Zn-MAX via the sublimation and volatilization of Zn at elevated temperatures, resulting in the transformation of the Zn-MAX phase to the TiC phase. Fe, Co, or Ni acetylacetone salts in NaCl-KCl molten salt bath were thermally decomposed, and transition metals were introduced into TiC lattice at elevated temperatures to occupy Ti vacancies. The strong polarization force of the NaCl-KCl molten salt bath effectively prevented the aggregation of transition metals into nanoclusters or metal nanoparticles. The final products were washed with 1 M HCl to remove residual Zn, and the obtained samples were named TiC-Fe_SA_, TiC-Co_SA_, and TiC-Ni_SA_.

**Figure 2:**
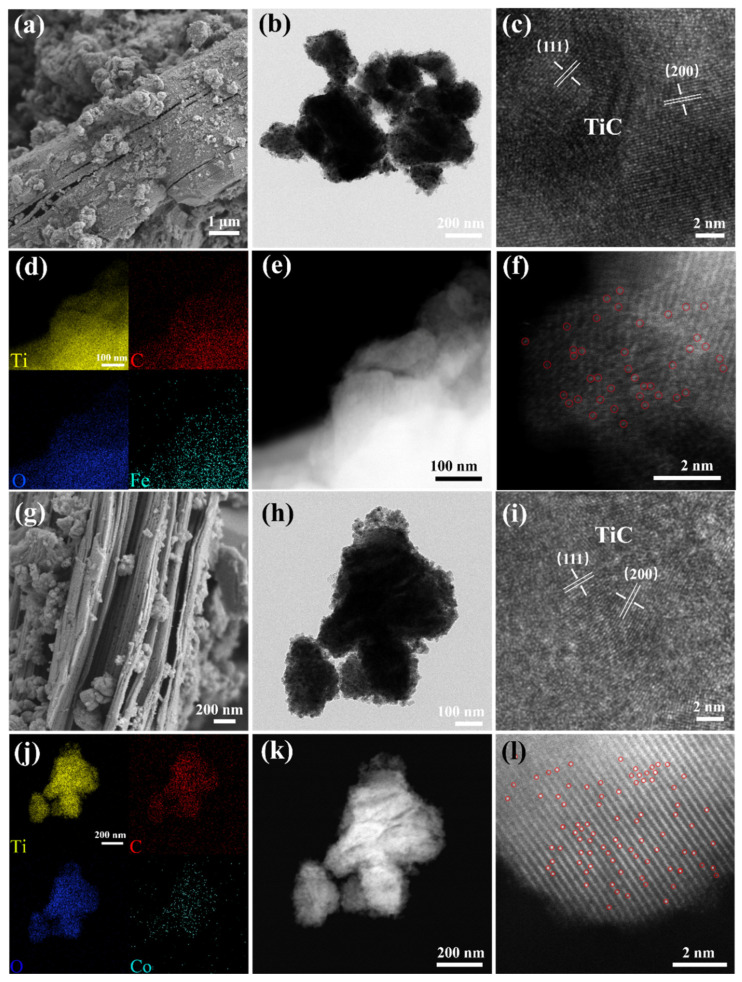
Characterization of structures of TiC-Fe_SA_ and TiC-Co_SA_. (**a**,**g**) SEM images and (**b**,**h**) low- and (**c**,**i**) high-magnification of TEM images of TiC-Fe_SA_ and TiC-Co_SA_, (**d**,**j**) corresponding EDS element mappings, (**e**,**k**) dark-field images of TiC-Fe_SA_ and TiC-Co_SA_, and (**f**,**l**) atomic-resolution HAADF-STEM images with Fe or Co single atoms highlighted in red circles.

**Figure 3:**
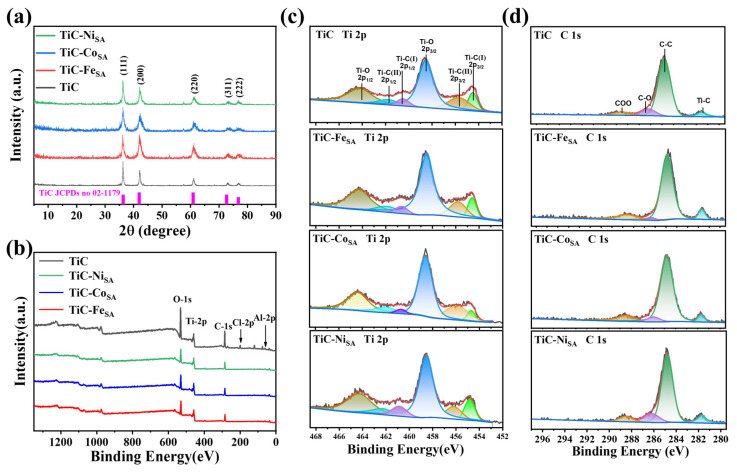
(**a**) XRD patterns of TiC, TiC-Fe_SA_, TiC-Co_SA_, and TiC-Ni_SA_. (**b**) The XPS survey spectra; (**c**) deconvoluted Ti 2p spectrum; and (**d**) deconvoluted C 1s spectrum of TiC, TiC-Fe_SA_, TiC-Co_SA_ and TiC-Ni_SA_.

The morphology of the samples was analyzed using a scanning electron microscope (SEM) and a transmission electron microscope (TEM). After Zn volatilization at high temperatures, the sample structure partially collapsed and exhibited lamellar stacking due to the lack of protection by the capping group and excessive calcination temperature [36] (Figure 2a,g, Figure S1a and Figures S2–S5). TiC nanosheet stacking can also be seen in TEM images (Figure 2b,i and Figure S1b). By measuring the lattice streaks in high-resolution images (Figure 2c,j and Figure S1f), distinct (111) and (200) crystal planes of TiC were observed, which were in agreement with the X-ray diffraction (XRD) results (Figure 3), and no metallic particle lattice of transition metals (Fe, Co, or Ni) were observed. In addition, the energy-dispersive spectroscopy (EDS) elemental mapping (Figure 2d,j and Figure S1d) of the samples showed a uniform distribution of Ti, C, O, and Fe, Co, or Ni, as well as the absence of transition-metal nanoparticles. Accordingly, a spherical aberration-corrected electron microscopy analysis of TiC-Fe_SA_ and TiC-Co_SA_, under a high-angle annular dark field, was performed to observe the distribution of Fe and Co. Relatively bright spots attributable to uniformly dispersed Fe or Co single atoms were observed, occupying Ti positions (red circles in Figure 2f,l), without obvious agglomeration of metal particles. Fe, Co, or Ni content in the samples was determined to be 0.166, 0.226, and 0.168 wt%, respectively, using inductively coupled plasma optical emission spectroscopy (ICP-OES). The very low metal content can be attributed to the low loading and effect of molten salt polarization, which ensures that the transition metals are uniformly dispersed in the form of single atoms without forming nanoclusters or nanoparticles [37,38].

The crystal structure of the samples was analyzed via XRD. The XRD pattern of Ti_2_ZnC showed a weak (002) peak, and the positions of (103) and (104) peaks shifted to a lower angle, indicating the successful substitution of Zn to Al in Ti_2_ZnC (Figure S6) [33]. The XRD patterns of the samples prepared via the high-temperature calcination of Zn-MAX phase showed the characteristic peaks of TiC, but no obvious peaks of Zn, Fe, Co, or Ni appeared, indicating that the metal particles were hardly formed after the volatilization of Zn at high temperatures and washing with HCl (Figure 3a). No XRD signals due to Fe, Co, Ni, and Zn were detected as a result of their low content, further indicating that the transition metals existed in the form of single atoms [19,39,40]. Next, the surface compositions of TiC, TiC-Fe_SA_, TiC-Co_SA_, and TiC-Ni_SA_ were analyzed using X-ray photoelectron spectroscopy (XPS). No obvious XPS peaks of transition metals were observed in the spectra of transition-metal-loaded TiC samples (Figure 3b and Figure S3), which may be due to the low metal loading below the detection limit of the instrument. Altogether, ICP-OES, XRD, XPS, and spherical aberration-corrected electron microscopy results demonstrated that Fe, Co, or Ni, are present in the samples at an atomic level. Figure 3c,d show the Ti 2p and C 1s high-resolution spectra of the samples. In the high-resolution Ti 2p spectrum of TiC, peaks located at 454.5 eV (460.5 eV) and 455.7 eV (461.8 eV) can be assigned to Ti-C (I) 2p_3/2_ (2p_1/2_) and Ti-C (II) 2p_3/2_ (2p_1/2_) [41,42], respectively. The 458.6 eV (464.2 eV) can be attributed to Ti-O 2p_3/2_ (2p_1/2_) [42,43]. The presence of O is due to the partial oxidation of the TiC surface during the washing and centrifugal drying of the sample. Meanwhile, the high-resolution C 1s spectra of TiC show peaks at 281.5, 284.8, 286.2, and 288.5 eV, which can be ascribed to Ti-C, C-C, C-O, and COO [41,42,44], respectively (Table S1).

### 2.2. Electrocatalytic Hydrogen Evolution Reaction Performance

**Figure 4:**
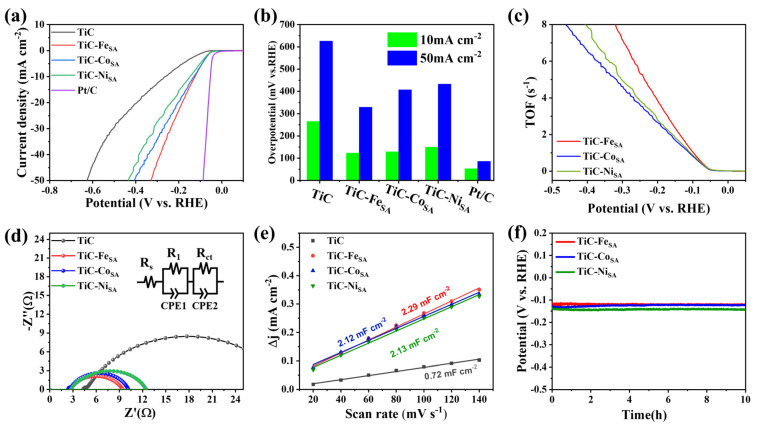
Hydrogen evolution performance of TiC-Fe_SA_, TiC-Co_SA_, and TiC-Ni_SA_ in 0.5 M H_2_SO_4_. (**a**) IR-corrected LSV polarization curves, (**b**) comparison of overpotentials at current densities of 10 and 50 mA cm^−2^, (**c**) hydrogen TOF curves, (**d**) equivalent circuit fitted Nyquist plots, (**e**) electrochemical double-layer capacitance calculated using the CV curves, and (**f**) chronopotentiometric curves at a current density of 10 mA cm^−2^.

To determine HER performance of TiC-Fe_SA_, TiC-Co_SA_, and TiC-Ni_SA_, linear sweep voltammetry (LSV) tests were performed in 0.5 M H_2_SO_4_ solution using a conventional three-electrode system. Figure 4a shows that the redox activity of samples was substantially enhanced after loading with Fe, Co, or Ni, and the overpotential decreased compared with pristine TiC in all cases. At 10 mA cm^−2^ current density, the overpotentials of pristine TiC and TiC-Fe_SA_ are 264.7 and 123.4 mV, showing the superior catalytic performance of Fe-loaded TiC (Table S2). Overpotentials of TiC-Co_SA_ and TiC-Ni_SA_ are 128.6 and 149.8 mV, respectively, considerably smaller than pristine TiC, proving that the introduction of transition metals successfully reduces HER overpotential and enhances the catalytic activity of TiC. Figure 4b shows the overpotentials at 10 and 50 mA cm^−2^ current densities. Among the three transition-metal-loaded TiC electrocatalysts, TiC-Fe_SA_ exhibits the lowest overpotential and the best HER performance, reaching 329.4 mV at 50 mA cm^−2^, which is lower than that of TiC-Co_SA_ (407.6 mV) and TiC-Ni_SA_ (432.6 mV). Assuming that the active centers of single atoms are all exposed to the electrolyte and that each active site is involved in the electrochemical reaction, the TOF values were calculated using the equation TOF = *j*A/2nF under these conditions [45], and the results are shown in Figure 4c. The TOF value of TiC-Fe_SA_ is 1.072 s^−1^ at an overpotential of 100 mV, which is higher than that of TiC-Co_SA_ (0.855 s^−1^) and TiC-Ni_SA_ (0.926 s^−1^), suggesting TiC-Fe_SA_ has higher intrinsic activity. The mass activity of the different electrocatalysts was obtained by normalizing the current density. As shown in Figure S4a, the mass activity of TiC-Fe_SA_ is 3.703 A mg^−1^ when the overpotential is 100 mV, which is higher than that of TiC-Co_SA_ (2.798 A mg^−1^) and TiC-Ni_SA_ (3.047 A mg^−1^), indicating that the catalytic activity of TiC-Fe_SA_ under acidic conditions is higher than that of TiC-Ni_SA_ and TiC-Co_SA_. Thus, Fe loading is more favorable for the HER under acidic conditions. Under acidic conditions, the HER process follows two steps: adsorption and desorption. Firstly, H ions react with electrons on the active site to form adsorbed H atoms (Volmer reaction). Then, depending on the kinetics, there are two ways of H desorption: two hydrogen atoms recombine and desorb (Tafel reaction), or adsorbed hydrogen combines with a hydrated hydrogen ion and electrons to produce hydrogen molecules (Heyrovsky reaction) [15]. The Tafel slope obtained from LSV curves is commonly used to evaluate HER kinetics, determine HER mechanism, and identify the rate-determining steps. When the Tafel slope value is more than 120 mV dec^−1^, the kinetic process is dominated by the Volmer reaction as the rate-determining step. The kinetic process using the Heyrovsky reaction as the rate-determining step has a Tafel slope ranging from 40 to 120 mV dec^−1^. When the Tafel process is the rate-determining step, the expected Tafel slope is 30 mV dec^−1^. [46]. To better understand the HER mechanism, Tafel plots were drawn using the polarization curves (Figure S5a). The Tafel slopes of TiC-Fe_SA_, TiC-Co_SA_, and TiC-Ni_SA_ are 61.1, 69.0, and 70.3 mV dec^−1^, respectively, which are considerably lower than that of pristine TiC (148.3 mV dec^−1^), indicating that loading with transition-metal single atoms promotes the reaction kinetics of HERs, thus enhancing the catalytic activity. The Tafel slope of TiC-Fe_SA_, TiC-Co_SA_, and TiC-Ni_SA_ are between 40 and 120 mV dec^−1^, indicating that the reaction process follows the Volmer-Heyrovsky mechanism dominated by the Heyrovsky reaction. In addition, to gain more insight into the electrochemical activity, the effect of the transition-metal loading on the charge transfer kinetics was investigated via electrochemical impedance spectroscopy (EIS). The data were fitted according to an equivalent circuit as depicted in Figure 4d, where *R*_s_, *R*_1_, and *R*_ct_ represent the internal resistance, solid electrolyte interface film resistance, and charge transfer resistance, respectively. CPE is the constant phase angle element, which represents the nonideal capacitance. Based on the fitted EIS data, the *R*_s_ of TiC, TiC-Fe_SA_, TiC-Co_SA_, and TiC-Ni_SA_ were 4.32, 2.43, 2.38, and 2.73 Ω, respectively, which were used for IR correction of LSV curves. The charge transfer resistances (*R*_ct_) of TiC-Fe_SA_, TiC-Co_SA_, and TiC-Ni_SA_ were determined to be 6.9, 7.5, and 8.7 Ω, respectively, which were lower than that of pristine TiC (26.2 Ω) (Figure 4d), indicating that the introduction of transition-metal single atoms successfully increases the charge transfer rate of TiC and accelerates the HER kinetics. As shown in Figure 4e, the double-layer capacitance (*C*_dl_) was calculated using cyclic voltammetry (CV) at 0.49–0.59 V with sweep rates ranging from 20 to 140 mV s^−1^. No redox reactions were observed in this range, and the current of the material was mainly due to the bilayer charging and discharging [47]. The *C*_dl_ value can be obtained using the slope of the fitting lines of the difference between half of the positive and negative scan current densities of the CV curve (*Δj*) obtained at different sweep speeds. The *C*_dl_ values of TiC-Fe_SA_, TiC-Co_SA_, and TiC-Ni_SA_ were 2.29, 2.12, and 2.13 mF cm^−2^, respectively, which were higher than those of pristine TiC (0.72 mF cm^−2^), indicating that the *C*_dl_ is considerably enhanced after the transition-metal loading, which exposes more active sites and thus promotes the HER kinetics. In addition to the catalytic activity, the stability of the hydrogen production is another important index to evaluate the performance of electrocatalysts. As shown in Figure S6a–c, the overpotentials of TiC-Fe_SA_, TiC-Co_SA_, and TiC-Ni_SA_ only decreased by about 9, 5, and 5 mV, respectively, at 10 mA cm^−2^ after 2000 CV cycles (Figure S11). The stability was also evaluated via constant current electrolysis at a current density of 10 mA cm^−2^ using the chronopotentionmetric method, and it was found that there was no substantial decrease in the overpotential after 10 h of constant current electrolysis (Figure 4f). The morphology of the electrocatalysts was observed via TEM after 10 h of constant current electrolysis. As shown by the TEM images in Figure S8a–c, the electrocatalysts maintained a lamellar stacked layer structure without obvious fragmentation after 10 h of constant current electrolysis, indicating that the transition-metal-loaded TiC showed excellent stability in long-term HERs.

**Figure 5:**
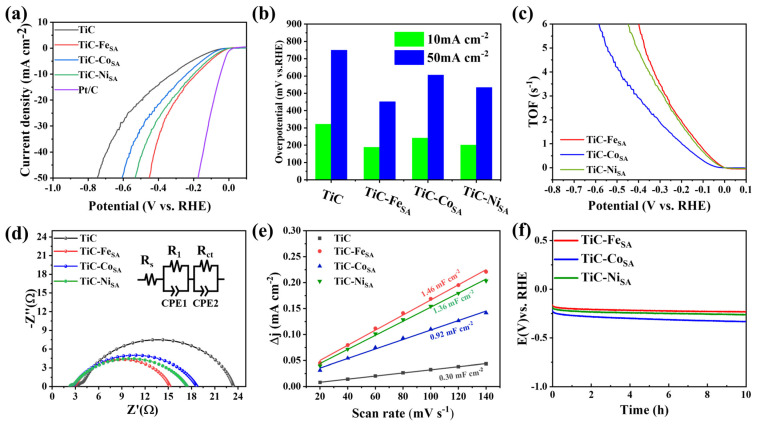
Hydrogen evolution performance of TiC-Fe_SA_, TiC-Co_SA_, and TiC-Ni_SA_ in 1 M KOH. (**a**) IR-corrected LSV polarization curves, (**b**) comparison of overpotentials at 10 and 50 mA cm^−2^, (**c**) hydrogen TOF curves, (**d**) equivalent circuit-fitted Nyquist plots, (**e**) electrochemical double-layer capacitance calculated using CV curves, and (**f**) chronopotentiometric curves at 10 mA cm^−2^ current density.

To investigate the performance of electrocatalysts under alkaline conditions, electrocatalysts were subjected to LSV measurements in 1 M KOH solution. Overpotentials of TiC-Fe_SA_, TiC-Co_SA_, and TiC-Ni_SA_ were considerably lower than those of pristine TiC (321.8 mV) (Figure 5a). The overpotential of TiC-Fe_SA_ (184.2 mV) was lower than that of TiC-Ni_SA_ (201.7 mV) and TiC-Co_SA_ (241 mV) at 10 mA cm^−2^ and was the lowest even at 50 mA cm^−2^ (Figure 5b and Table S2). To further investigate the catalytic activity under alkaline conditions, the TOF values were calculated. TiC-Fe_SA_ exhibited the highest TOF value of 0.313 s^−1^ at an overpotential of 50 mV under alkaline conditions compared with TiC-Ni_SA_ (0.253 s^−1^) and TiC-Co_SA_ (0.074 s^−1^), indicating that TiC-Fe_SA_ showed the highest catalytic activity (Figure 5c). The mass activity was calculated by normalizing the current density after the transition-metal single-atom loading (Figure S4b). The mass activity of TiC-Fe_SA_ was 2.789 A mg^−1^ when the overpotential was 100 mV, higher than TiC-Co_SA_ (1.056 A mg^−1^) and TiC-Ni_SA_ (2.194 A mg^−1^), indicating that Fe loading is more favorable for HERs under alkaline conditions and resulting in a better catalytic performance. Under alkaline conditions (Figure S5b), Tafel slopes were determined to be 85.1, 95.5, and 93 mV dec^−1^ for TiC-Fe_SA_, TiC-Co_SA_, and TiC-Ni_SA_, respectively, which were lower than pristine TiC (141 mV dec^−1^), indicating that the transition-metal single-atom loading, particularly Fe loading, promotes HER kinetics under alkaline conditions. The HERs of TiC-Fe_SA_, TiC-Co_SA_, and TiC-Ni_SA_ still followed the Volmer-Heyrovsky mechanism dominated by the Heyrovsky reaction. The electron transfer efficiency of electrocatalysts was evaluated by measuring the electrochemical impedance of electrocatalysts under alkaline conditions. Figure 5d shows that the *R*_s_ of TiC, TiC-Fe_SA_, TiC-Co_SA_, and TiC-Ni_SA_ are 3.34, 2.58, 2.39, and 2.30 Ω, respectively, which are used for IR correction. The *R*_ct_ values of TiC-Fe_SA_, TiC-Co_SA_, and TiC-Ni_SA_ were 12.27, 15.99, and 14.79 Ω, respectively. The charge transfer resistance of the transition-metal-loaded TiC electrocatalysts decreased compared to pristine TiC (19.1 Ω). TiC-Fe_SA_ exhibits the lowest charge transfer resistance, highest charge transfer efficiency, and best electrical conductivity. *C*_dl_ of electrocatalysts was calculated by fitting the measured CV curves under alkaline conditions at different sweep speeds ranging between 20 and 140 mV s^−1^ within a limited scan range of 0.75–0.85 V. *C*_dl_ values of TiC-Fe_SA_ (1.46 mF cm^−2^), TiC-Ni_SA_ (1.36 mF cm^−2^), and TiC-Co_SA_ (0.92 mF cm^−2^) are higher than pristine TiC 0.30 mF cm^−2^), indicating that the (transition-metal-loaded TiC, especially TiC-Fe_SA_, can expose more active sites to promote HER (Figure 5e). The results indicate that TiC-Fe_SA_ exhibited the highest HER activity under alkaline conditions with the lowest elemental loading, and its catalytic performance was considerably higher than that of TiC-Co_SA_ and TiC-Ni_SA_. Next, the stability of the electrocatalysts was analyzed using LSV measurements after 2000 cycles of CV (Figure S7a–c). Overpotentials of TiC-Fe_SA_, TiC-Co_SA_, and TiC-Ni_SA_ did not decrease substantially at 10 mA cm^−2^ and decreased by approximately 19.9, 15.2, and 24.9 mV at 50 mA cm^−2^ (Figure S14). In a chronopotentiometric test under constant current electrolysis of 10 mA cm^−2^ (Figure 5f), electrocatalysts maintained high stability for 10 h without any remarkable drop in their overpotential, indicating their high durability. TEM images recorded after 10 h of constant current electrolysis (Figure S8d–f) showed that the electrocatalysts maintained their complete flake morphology without obvious oxidative fragmentation, indicating that they remain highly stable after 10 h of constant current electrolysis tests. 

### 2.3. Density Functional Theory Calculations

**Figure 6:**
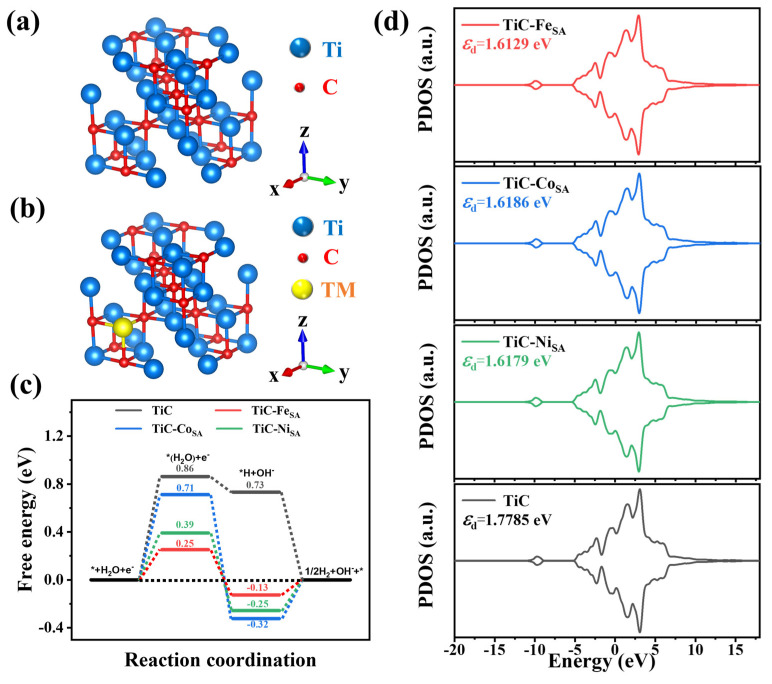
Crystal structures of (**a**) TiC and (**b**) TiC-TM (TM = transition metal). (**c**) Hydrolysis dissociation barriers and hydrogen adsorption-free energies calculated for pristine TiC and TiC-Fe_SA_, TiC-Co_SA_, and TiC-Ni_SA_, where * represents the active site. (**d**) The d-orbital localized density of states (d-LDOS) of pristine TiC, TiC-Fe_SA_, TiC-Co_SA_, and TiC-Ni_SA_.

To understand the effect of the single-atom loading on the catalytic performance of TiC, DFT calculations were systematically performed using TiC models with Fe, Co, or Ni atoms occupying one Ti atom position as the active adsorption site (Figure 6a,b). The closer the hydrogen adsorption Gibbs free energy (ΔG_H*_) is to zero, the more favorable it is for HERs to proceed [48,49]. Pristine TiC adsorbs hydrogen at a ΔG_H*_ of approximately 0.73 eV, which suggests a weak binding between Ti and H (Figure 6c). ΔG_H*_ of transition-metal-loaded TiC decreases, with TiC-Fe_SA_ being the closest to 0 (−0.13 eV) compared with TiC-Co_SA_ (−0.32 eV) and TiC-Ni_SA_ (−0.25 eV), respectively, which suggests that Fe single-atom loading is more favorable for hydrogen adsorption and HER under acidic conditions. In contrast, during alkaline HERs, the required protons primarily come from water dissociation. Enhanced water adsorption is favorable for the Volmer and Heyrovsky steps under alkaline conditions [50,51]; thus, water adsorption plays an important role. A comparison between the water adsorption Gibbs free energy (ΔG_H2O*_) and ΔG_H*_ shows that the former was greater than the latter, suggesting that the water adsorption step was the rate-controlling step of HERs under alkaline conditions. Pristine TiC had the largest ΔG_H2O_ of 0.86 eV compared with the values of 0.25, 0.71, and 0.39 eV for TiC-Fe_SA_, TiC-Co_SA_, and TiC-Ni_SA_, respectively. The results show that loading with transition-metal single atoms, particularly Fe loading, effectively improves the water absorption capacity of TiC and HER kinetics under alkaline conditions. According to the d-band theory, a position closer to zero for the d-band center would lead to stronger binding interactions between the catalyst and adsorbate [52,53,54], which enhances hydrogen adsorption. A projected state density analysis of TiC was performed for pristine TiC and transition-metal-loaded TiC electrocatalysts. The d-band centers of TiC-Fe_SA_, TiC-Co_SA_, and TiC-Ni_SA_ were all shifted toward the Fermi energy level compared with pristine TiC, indicating that loading with transition-metal single atoms, especially Fe, is more favorable for hydrogen adsorption, which is consistent with the results of ΔG_H*_ calculations (Figure 6d).

## 3. Materials and Methods

### 3.1. Preparation of Ti_2_ZnC

Zn-MAX phase was synthesized based on a reported procedure [33]. Briefly, Ti_2_AlC was mixed with ZnCl_2_ in a molar ratio of 1:2 in a mortar and ground evenly, and the resulting mixture was put into an alumina porcelain boat, which was then placed in a tube furnace and heated at 550 °C under flowing Ar protection for 5 h. The product was washed with deionized water to remove the residual ZnCl_2_ and then dried at 60 °C.

### 3.2. Preparation of TiC

A mixture of 1 g Ti_2_ZnC using 1.692 g of NaCl and 2.1581 g of KCl (1:5:5 molar ratio) was put into a mortar and ground evenly. The resulting mixture was put into an alumina porcelain boat and taken to a tube furnace, where it was heated at 950 °C for 2 h under the protection of flowing Ar. The product was washed with 1 M HCl to remove residual Zn particles and freeze-dried.

### 3.3. Preparation of TiC-Fe_SA_, TiC-Co_SA_, and TiC-Ni_SA_

A porcelain boat containing 0.1 g of the corresponding acetylacetonate salt (iron acetylacetonate, cobalt acetylacetonate, or nickel acetylacetonate) was placed upstream of a porcelain boat containing Zn-MAX-mixed molten salt, and the rest of the steps were the same as those described for the preparation of TiC. The obtained samples were named as TiC-Fe_SA_, TiC-Co_SA_, and TiC-Ni_SA_.

## 4. Conclusions

TiC electrocatalysts loaded with transition-metal single atoms (Fe, Co, or Ni) were synthesized using the molten salt method. By introducing transition-metal single atoms, the electronic structure of the active center was adjusted. The transition-metal loadings of TiC-Fe_SA_, TiC-Co_SA_, and TiC-Ni_SA_ were as low as 0.166, 0.226, and 0.168 wt%, respectively, but they were sufficient to decrease the overpotential and enhance the catalytic activity of TiC. At 10 mA cm^−2^ current density, overpotentials of TiC-Fe_SA_, TiC-Co_SA_, and TiC-Ni_SA_ were 123.4, 128.6, and 149.8 mV under acidic conditions and 184.2, 241, and 201.7 mV under alkaline conditions. The electrocatalysts maintained good long-term stability under acidic and alkaline conditions. The mass activity and TOF calculations show that loading Fe into the TiC matrix enhances HER performance more than Ni and Co under acidic and alkaline conditions. DFT calculations show that the introduction of Fe, Co, or Ni single atoms into TiC can effectively reduce the reaction energy barriers of TiC electrocatalysts, especially in the case of Fe. 

## Data Availability

The data presented in this study are available on request from the corresponding author.

## Supplementary Materials

The following supporting information can be downloaded at: https://www.mdpi.com/article/10.3390/ijms25074034/s1. Additional structural characterizations and performance tests of the catalysts. DFT computational methods and related references [55,56,57,58,59,60,61,62,63].
