# Supplementary material for: Loading of Single Atoms of Iron, Cobalt, or Nickel to Enhance the Electrocatalytic Hydrogen Evolution Reaction of Two-Dimensional Titanium Carbide
Source: Int J Mol Sci. 2024 Apr 4;25(7):4034. doi: 10.3390/ijms25074034 (PMC11012987; doi:10.3390/ijms25074034)
Supplement: Supplementary file 1 [file ijms-25-04034-s001.zip › ijms-2946846-supplementary.pdf]

Supporting Information for:

Loading of Single Atoms of Iron, Cobalt, or Nickel to Enhance the Electrocatalytic Hydrogen Evolution Reaction of Two-Dimensional Titanium Carbide

Kaijin Wang, Jing Yu*, Qi Liu, Jingyuan Liu, Rongrong Chen, Jiahui Zhu, and Jun Wang

Key Laboratory of Superlight Materials and Surface Technology, Ministry of Education, College of Materials Science and Chemical Engineering,

Harbin Engineering University, Harbin 150001, China

*E-mail: jing.yu@hrbeu.edu.cn.

Characterizations

The Rigaku TTRIIIX instrument used for the X-ray diffractometer (XRD) has a scan angle 2θ range of 5°-90°, a radiation source of Cu Kα radiation (λ=1.5418 Å), an operating current of 150 mA, an operating voltage of 40 kV, and a scan speed of 10°/min. Transmission electron microscopy (TEM) and X-ray spectroscopy elemental mapping (EDS) were performed using the FEI Talos F200X TEM at 200 kV. XPS data were performed on X-ray photoelectron spectra (ESCALAB 250Xi, *hv*= 1486.6 eV, Al Kα radiation) and corrected by the C1s standard peak (284.8 eV). The sample transition metal monoatomic content was obtained by ICP-OES testing.

Electrochemical measurements

Electrochemical measurements are performed in a standard three-electrode system using a CHI660E electrochemical workstation with a pt-sheet as counter electrode and a saturated glymeric electrode as reference electrode, with the working electrode prepared in the well-known manner. First, an aqueous solution of catalyst ink was prepared by homogeneously dispersing 5 mg of catalyst in 250 μL of water, 250 μL of ethanol and 30 μL of Nafion (5 wt%). After sonicating the ink for half an hour, 100 μL of the ink-water solution was evenly added dropwise onto a carbon cloth with an area of 1 cm*1 cm and dried for 40 minutes to produce a working electrode.

All electrochemical tests were calibrated by reversible hydrogen electrode (E_{RHE}=E_{SCE}+0.0591×pH+0.241). After activating the electrode with CV, the LSV polarization curve was tested under 0.5 M H_2SO_4 aqueous electrolyte with a scan rate of 2 mV/s and corrected by 95% *IR* compensation. The AC impedance spectrum (EIS)

is tested in the frequency range of 10^5 Hz to 10^{-1} Hz. The CV curves were measured by sweeping at 20, 40, 60, 80, 100 and 120 mV/s, and the CV curves were used to fit the calculation of the bilayer capacitance (C_{dl}). The equation Tof =jA/2nf was applied to calculate the amount of hydrogen converted per unit time at each active site of the catalyst [45]. j is the current density, obtained by LSV test. A is the electrode surface area (1 cm*1 cm). 2 is the transfer of 2 mol of electrons per 1 mol of H^2 produced in the hydrogen precipitation reaction. n is the molar fraction of active centers (calculated by ICP test), and F is the Faraday constant (96485 C mol^{-1}).

Computational Methods

All the DFT calculations were performed using the Vienna ab initio Simulation Package (VASP) [55-57]. The projector augmented wave (PAW) pseudopotential with a cutoff energy of 600 eV was adopted to describe the core electrons , and the Perdew-Burke-Ernzerhof (PBE) exchange-correction functional treated by generalized gradient approximation (GGA) was employed to deal with the electron interactions [58-60]. Partial occupancies of the Kohn−Sham orbitals were allowed using the Gaussian smearing method and a width of 0.05 eV. The electronic energy was considered self-consistent when the energy change was smaller than 10^{-6} eV. A geometry optimization was considered convergent when the energy change was smaller than 0.05 eV/Å. The brillouin zone is sampled with 4 × 4 × 3 Monkhorst mesh [61].

For acidic HER in the Volmer-Tafel mechanism, the elementary reactions include two processes as follows [62]:

$H_3O^+ + e^- + ^* \rightarrow H^* + H_2O$ (S1)

$H^* + H^* \rightarrow H_2 + 2^*$ (S2)

For alkaline HER in the Volmer-Tafel mechanism, the elementary reactions include four processes as follows [63]:

$H_2O + ^* \rightarrow H_2O^*$ (S3)

$H_2O^* \rightarrow H^* + OH^*$ (S4)

$H^* + OH^* + e^- \rightarrow H^* + OH^-$ (S5)

$H^* \rightarrow \ ^* + 1/2H_2$ (S6)

The reaction free energy of each elementary step can be calculated using the following equation:

$\Delta G = \Delta E + \Delta ZPE - T\Delta S$ (S7)

where ΔE is the electronic energy difference directly available from DFT computation, ΔZPE is the correction in zero-point energies (*ZPE*), T is the room temperature (here T = 298.15 K), ΔS is the entropy change.

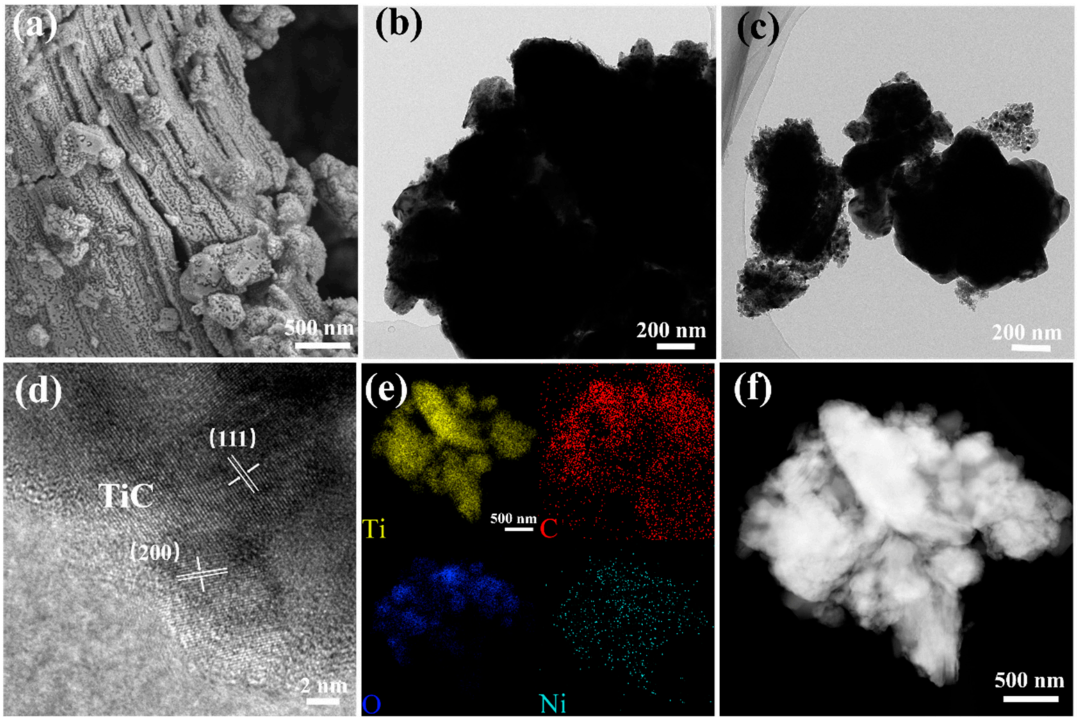

Figure S1. Characterization of the structure of TiC-Ni_{SA}. (a) SEM image and TEM images at low (b,c) and high (d) magnifications of TiC-Ni_{SA}. (e) Corresponding EDS element mappings. (f) Dark field images of TiC-Ni_{SA}.

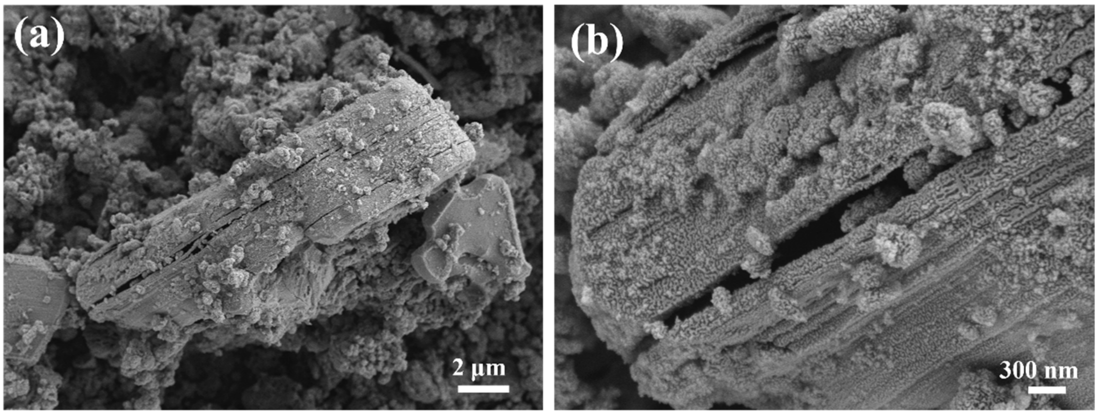

Figure S2. SEM images of TiC-Fe_{SA}.

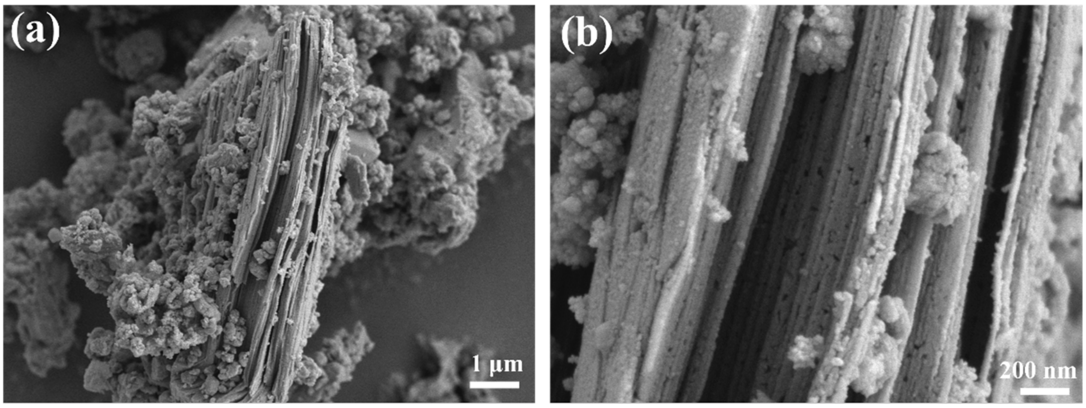

Figure S3. SEM images of TiC-Co_{SA}.

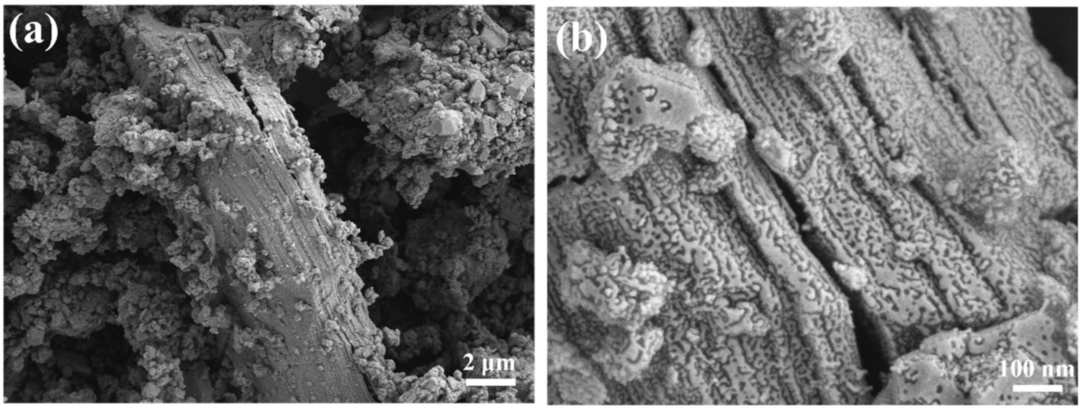

Figure S4. SEM images of TiC-Ni_{SA}.

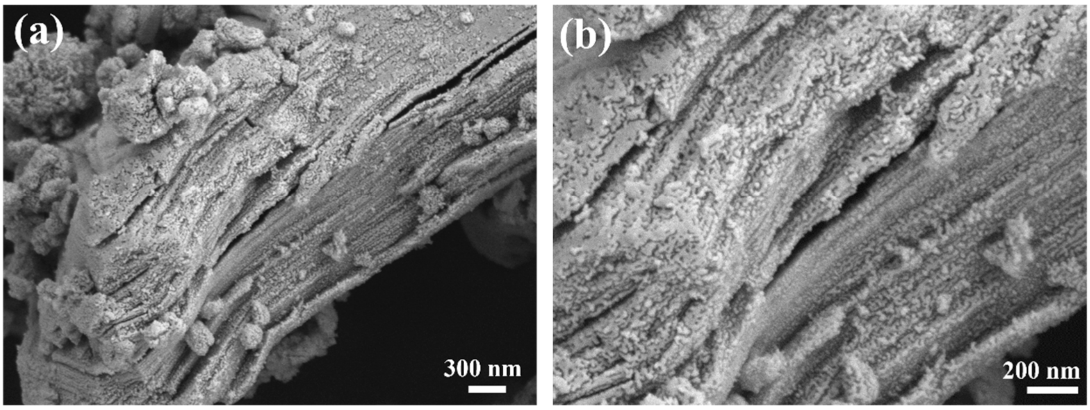

Figure S5. SEM images of initial TiC.

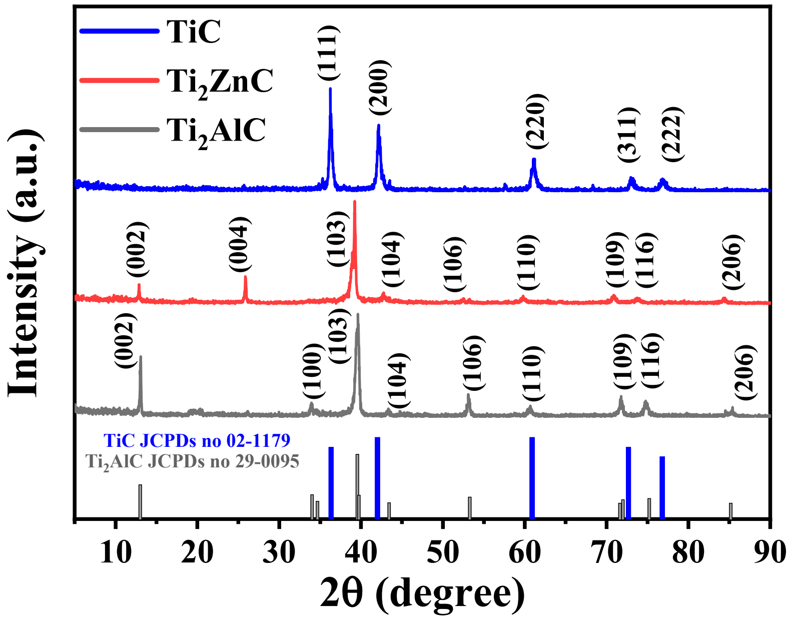

Figure S6. XRD spectra of Ti_2AlC, Ti_2ZnC and TiC.

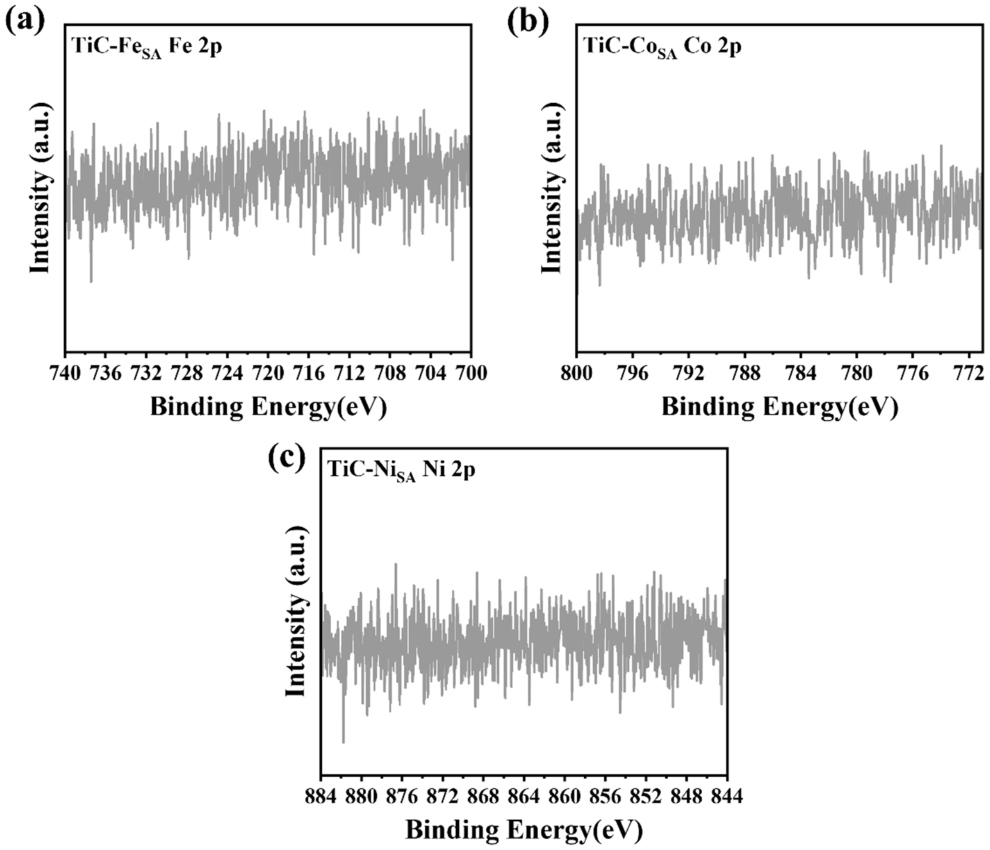

Figure S7. Deconvoluted Fe 2p(a), Co 2p(b) and Ni 2p (c) XPS spectra.

Table S1. XPS peak fitting of results of sample.

		TiC	TiC-Fe_{SA}	TiC-Co_{SA}	TiC-Ni_{SA}
Ti $2p_{3/2}$ ($2p_{1/2}$)	Ti-C	454.5 eV (460.5 eV)	454.6 eV (460.6 eV)	454.7 eV (460.7 eV)	454.8 eV (460.8 eV)
	Ti-C	455.7 eV (461.8 eV)	455.8 eV (461.9 eV)	455.9 eV (462.0 eV)	456.2 eV (462.3 eV)
	Ti-O	458.5 eV (464.2 eV)	458.5 eV (464.3 eV)	458.6 eV (464.4 eV)	458.6 eV (464.3 eV)
C 1s	Ti-C	281.5 eV	281.6 eV	281.7 eV	281.7 eV
	C-C	284.8 eV	284.8 eV	284.8 eV	284.8 eV
	C-O	286.2 eV	286.2 eV	286.2 eV	286.2 eV
	COO	288.5 eV	288.5 eV	288.5 eV	288.5 eV

Table S2. The comparison of HER performance upon the as-prepared samples in 0.5 M H_2SO_4 and 1 M KOH.

Electrocatalysts	Overpotential at 10 mA cm^{-2} (mV, 0.5 M H_2SO_4)	Overpotential at 10 mA cm^{-2} (mV, 1 M KOH)
TiC	264.7	321.8
TiC-Fe_{SA}	123.4	184.2
TiC-Co_{SA}	128.6	241.0
TiC-Ni_{SA}	149.8	201.7

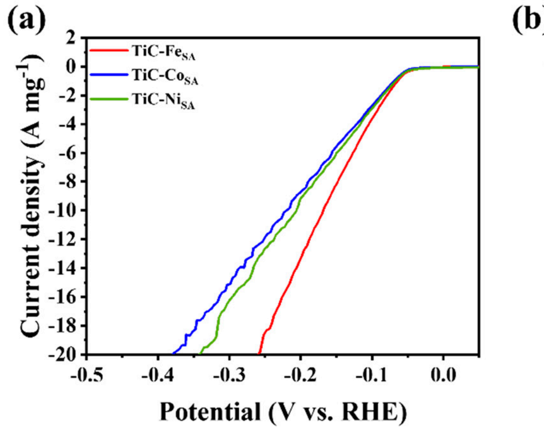

Figure S8. Plots of mass activity of TiC samples loaded with different transition metal single atoms in 0.5 M H_2SO_4 (a) and 1 M KOH (b).

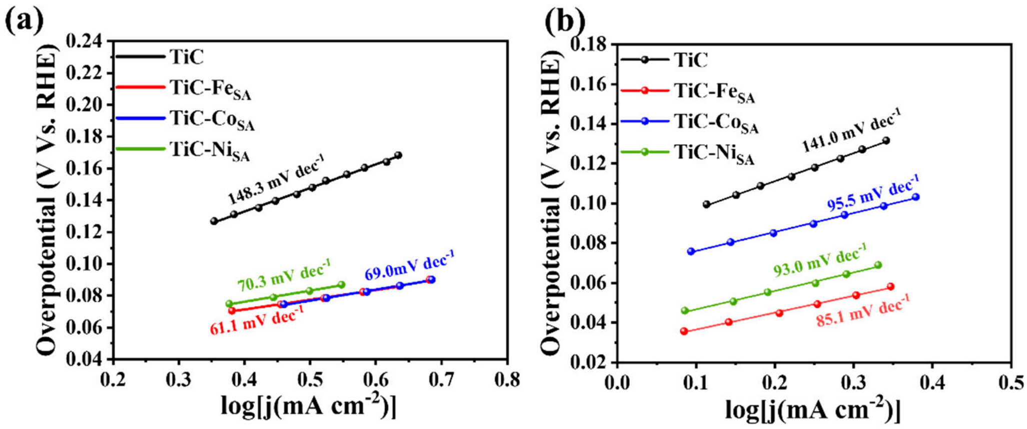

Figure S9. Tafel slope plots of TiC samples loaded with different transition metal single-atoms under acidic (a) and alkaline (b) conditions.

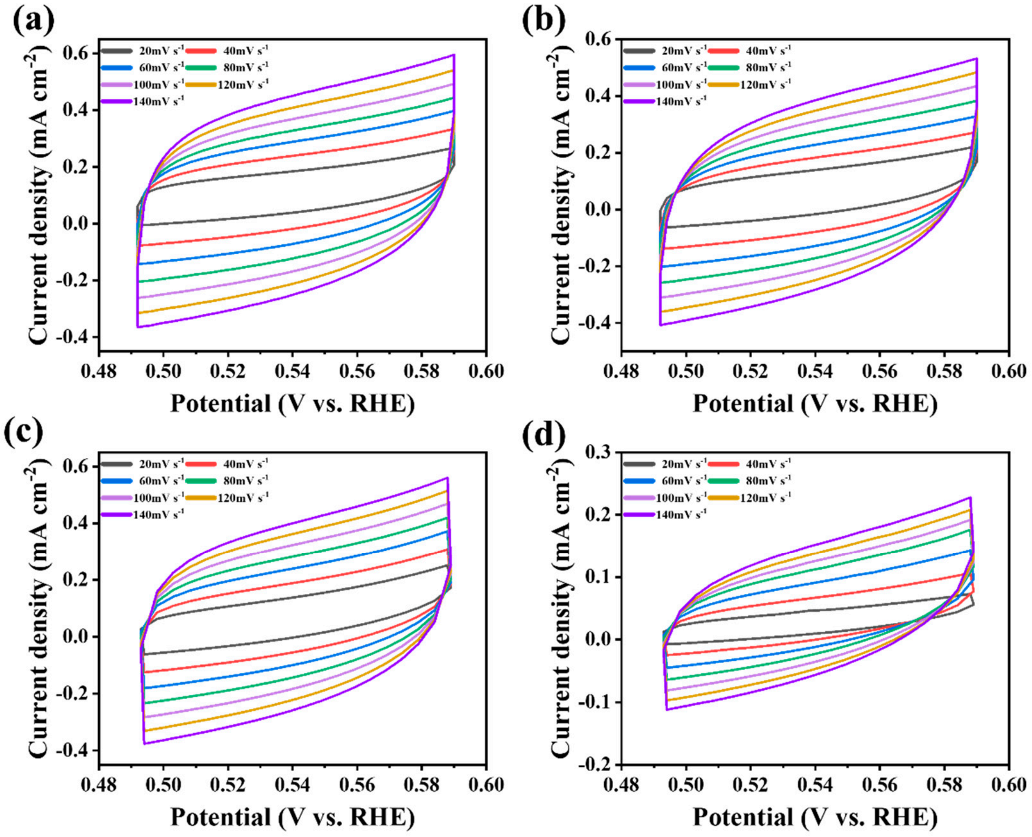

Figure S10. CV curves from different scan rates from 20 to 140 mV s^{-1} in 0.5 M H_2SO_4 for HER.

(a) TiC-Fe_{SA}, (b) TiC-Co_{SA}, (c) TiC-Ni_{SA}, and (d) TiC.

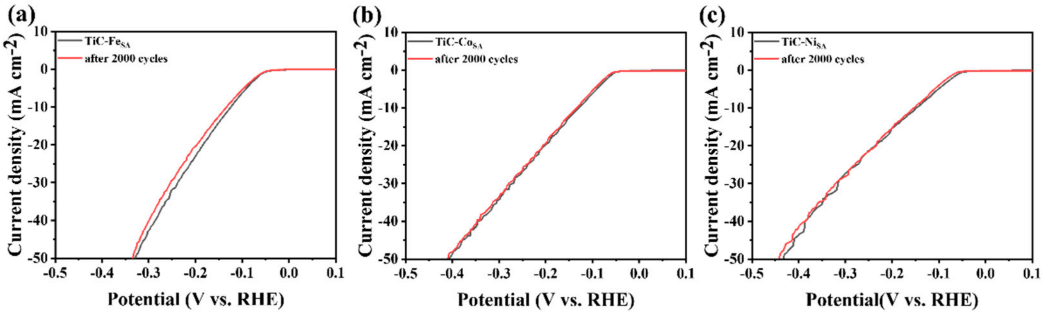

Figure S11. Polarization curves of LSV of TiC loaded with different transition metal single-atoms before and after 2000 CV cycles under acidic conditions.

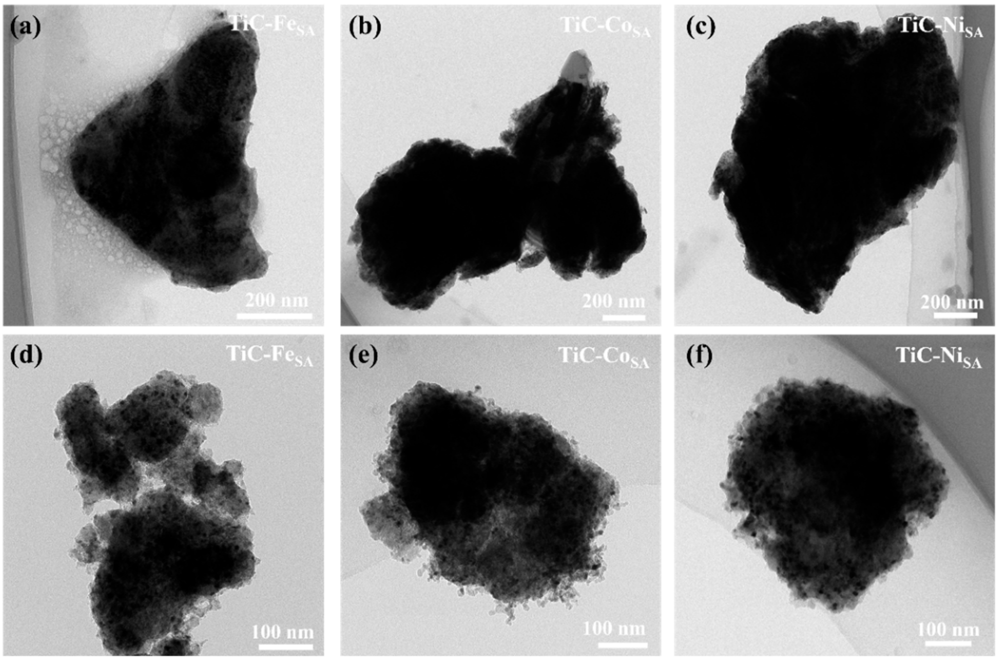

Figure S12. TEM images of TiC with different monoatomic loadings after 10 hours of constant current electrolysis at 10 mA cm^{-2} current density under acidic (a-c) and alkaline (d-f) conditions.

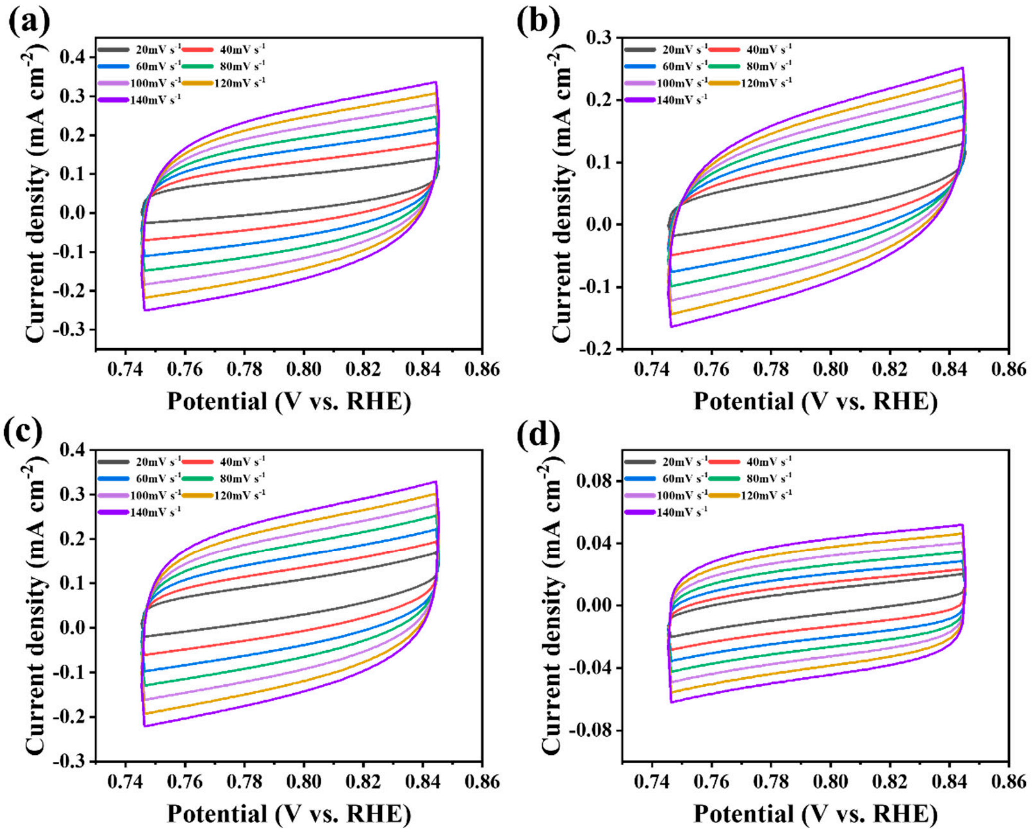

Figure S13. CV curves from different scan rates from 20 to 140 mV s^{-1} in 1 M KOH for HER. (a) TiC-Fe$_{SA}$, (b) TiC-Co$_{SA}$, (c) TiC-Ni$_{SA}$, and (d) TiC.

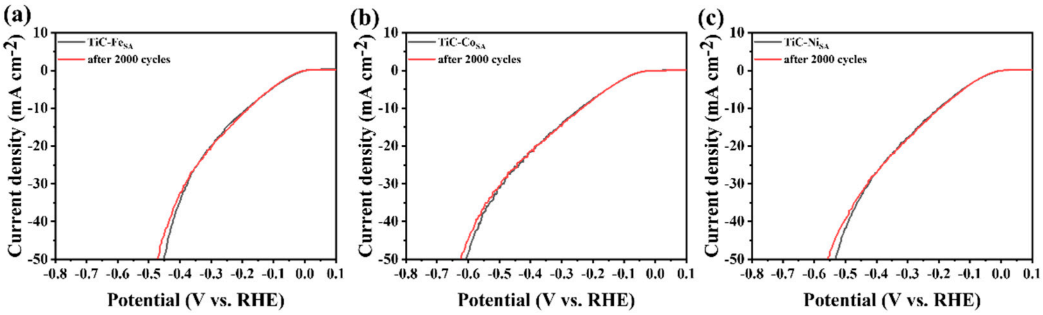

Figure S14. Polarization curves of LSV of TiC loaded with different transition metal single-atoms before and after 2000 CV cycles under alkaline conditions.

References

45. Shi, Y.; Ma, Z.R.; Xiao, Y.Y.; Yin, Y.C.; Huang, W.M.; Huang, Z.C.; Zheng, Y.Z.; Mu, F.Y.; Huang, R.; Shi, G.Y.; et al. Electronic Metal-Support Interaction Modulates Single-Atom Platinum Catalysis for Hydrogen Evolution Reaction. *Nat. Commun.* **2021**, 12, 3021.

55. Kresse, G.; Hafner, J.; Ab Initio Molecular Dynamics for Liquid Metals. *Phys. Rev. B* **1993,** 47, 558-561.

56. Kresse, G.; Hafner, J.; Ab Initio Molecular-Dynamics Simulation of the Liquid-Metalamorphous-Semiconductor Transition in Germanium. *Phys. Rev. B* **1994,** 49, 14251-14269.

57. Liu, J.; Xiao, J.; Luo, B.; Tian, E.; Waterhouse, G.I.N.; Central Metal and Ligand Effects on Oxygen Electrocatalysis over 3dS Transition Metal Single-Atom Catalysts: A Theoretical Investigation. *Chem. Eng. J.* **2022,** 427. 132038.

58. Blöchl, P.E.; Projector Augmented-Wave Method. *Phys. Rev. B* **1994,** 50, 17953-17979.

59. Kresse, G.; From Ultrasoft Pseudopotentials to the Projector Augmented-Wave Method. *Phys. Rev. B* **1999,** 59, 1758-1775.

60. Perdew; Burke; Ernzerhof; Generalized Gradient Approximation Made Simple. *Phys. Rev. Lett.* **1996,** 77, 3865-3868.

61. Norskov, J.K.; Rossmeisl, J.; Logadottir, A.; Lindqvist, L.; Kitchin, J.R.; Bligaard, T.; Jonsson, H.; Origin of the Overpotential for Oxygen Reduction at a Fuel-Cell Cathode. *J. Phys. Chem. B* **2004,** 108, 17886-17892.

62. Deng, J.; Ren, P.; Deng, D.; Yu, L.; Yang, F.; Bao, X.; Highly Active and Durable Non-Precious-Metal Catalysts Encapsulated in Carbon Nanotubes for Hydrogen Evolution Reaction. *Energy Environ. Sci.* **2014,** 7, 1919-1923.

63. Liu, X.; Jiao, Y.; Zheng, Y.; Davey, K.; Qiao, S.-Z.; A Computational Study on Pt and Ru Dimers Supported on Graphene for the Hydrogen Evolution Reaction: New Insight into the Alkaline Mechanism. *J. Mater. Chem. A* **2019,** 7, 3648-3654.
